# On Various Uses of Collargol

**Published:** 1909-04

**Authors:** 


### On Various Uses of Collargol

### COLLARGOL IN SURGERY.

PROFESSOR ROSWELL PARK (Principles and Practice of Modern Surgery") says regarding the treatment of septicemia: "Of great value also will be found the silver ointment of Credé (unguentum Credé). This permits of absorption of silver through the unbroken skin (as in the case of ung. hydrarg.) and the dissemination throughout the system of the antiseptic virtue of the silver itself. To ensure its greatest efficacy, this ointment should be thoroughly rubbed in, especially over parts which are not too tender. Many cases of septic infection promptly yield under the influence of the argentine preparations which Credé has lately introduced. Another measure of great utility in selected cases is the intravenous infusion of a solution of Credé's soluble silver, made with 1 gram of silver in 1000 c.c. of sterilised water at a temperature of 105° to 110°. In cases of profound toxemia a small amount of blood may be withdawn (50 to 400 c.c.) No hesitation need be felt in introducing 500 or even 1000 c.c. of this solution. It is the ideal way of bringing a powerful non-toxic antiseptic into immediate contact with pathogenic microbes."

### COLLARGOL TO CHILDREN.

Professor B. K. Rachford ("Administration of Drugs to Children by Inunction;" (*American Journal of Medical Sciences*, January, 1909), says that "Colloidal silver within the past few years has been administered hypodermatically by the stomach and by inunction in the treatment of various forms of localised and general septicemia. The profession, as a whole, I think, has come to recognise that this is a most valuable adjunct in our treatment of septicemia, and I for one, after a large experience extending over quite a number of years, am firmly convinced of its efficacy. In the acute enlargement of the lymphatic tissues of the neck which may follow scarlatinal, diphtheritic and other forms of tonsilitis, I believe that this remedy, in the form of unguentum Credé, properly rubbed into the surrounding lymphatic tissues, is of very great value in preventing the spread of the disease and in controlling the localised sepsis. This drug can be given more efficaciously to infants and young children by inunction than in any other manner, and its value in combating general and localised sepsis is of much more value in infants and children than it is in adults, because inunctions in general are more efficacious in children than they are in adults."

### COLLARGOL IN JOINT RHEUMATISM.

Professor Plehn (Municipal Hospital Urban, at Berlin. *Deutsche med. Wochenschrift*, December 24, 1908), says that "Our most approved remedy in joint rheumatism is collargol, intravenously as recommended by Credé, and as systematically employed by Riebold in the Dresden Municipal Hospital. The attempts at explaining the curative processes are as hypothetical as with salicylic acid; development of the disease germs is said to be hindered, a leukocytosis stimulated, catalytic processes set up, and the like. But, be this as it may, the favorable effect is hardly to be doubted, and for some time past we have not hesitated to use collargol intravenously in joint rheumatism which is exceptionally resistant to salicylic acid, or when salicylic is not tolerated well enough to be given in full doses, or when the discontinuance of salicylic acid is followed by repeated relapses."

COLLARGOL IN CYSTITIS.

PROFESSOR JEANBRAU (*Montpelier Medical*, May 31, 1908), says he had his attention called to the use of collargol in cystitis by Stoos and Tavel at the Berne Infant Hospital. His result in all cases was the same as from silver nitrate. But the great advantage of collargol over silver nitrate consists in its painlessness. In acute cystitis, the nitrate is agonising; here collargol is as effective without causing pain. Collargol quickly inhibits the pain of cystitis.

